# Risk Factors and Vascular Features Associated With Local Recurrence in Pancreatic Cancer Post‐Pancreaticoduodenectomy: A Retrospective Cohort Study

**DOI:** 10.1002/cnr2.70267

**Published:** 2025-07-01

**Authors:** Ting‐Kai Liao, Ying Jui Chao, Wei‐Hsun Lu, Ping‐Jui Su, Chih‐Jung Wang, Yan‐Shen Shan

**Affiliations:** ^1^ Department of Surgery National Cheng Kung University Hospital, College of Medicine, National Cheng Kung University Tainan Taiwan; ^2^ Institute of Clinical Medicine, College of Medicine, National Cheng Kung University Tainan Taiwan

**Keywords:** local recurrence, pancreatic cancer, pancreaticoduodenectomy, portal vein resection, risk factors, vascular features

## Abstract

**Objectives:**

This study aims to analyze the risk factors for local recurrence (LR) following pancreaticoduodenectomy (PD) in pancreatic cancer patients and to identify vascular features associated with this outcome.

**Background:**

Pancreatic cancer frequently involves the mesenteric root, particularly the Porto‐mesenteric vein (PV‐SMV), impacting survival post curative surgery. However, the relationship between vascular structural changes and LR post‐operation remains unclear.

**Methods:**

Retrospective data collection was conducted at a single tertiary center from December 2010 to March 2021. Clinical characteristics, surgical‐pathological factors, and radiological features were compiled.

**Results:**

A total of 203 pancreatic cancer cases undergoing PD were analyzed, with 72 (35.5%) undergoing concurrent PV‐SMV resection (VR). Median overall survival post‐operation was 22.4 months. LR occurred in 121 patients (60%) at a median time of 8 months postoperatively. Resectable disease exhibited significantly longer local‐recurrence free survival compared to borderline resectable/locally advanced pancreatic cancer (BRPC/LAPC) (median 14.5 vs. 7 months, *p* < 0.001). The most frequent sites of LR were the mesenteric root (37%), superior mesenteric artery (SMA, 21%), and superior mesenteric vein (SMV, 16%), with similar patterns observed in the VR and non‐VR groups. BRPC, LAPC, postoperative CA19‐9 above normal range, venous thrombosis, and stenosis were associated with LR (HR: 2.1 [95% CI 1.21–3.68], 2.7 [95% CI 1.6–4.71], 1.8 [95% CI 1.21–2.69], 2.0 [95% CI 1.08–3.92], and 1.6 [95% CI 1.0–2.65], respectively), while PV‐SMV resection and enlargement of PV‐SMV angle were protective factors (HR: 0.4 [95% CI 0.25–0.67] and 0.3 [95% CI 0.19–0.53]).

**Conclusions:**

Despite aggressive treatment strategies including neoadjuvant therapy and radical surgery, LR in pancreatic cancer remains a challenge. This study highlights potential risk factors, recurrence patterns, and associated vascular features for early identification. These findings may guide clinicians in developing more targeted surveillance strategies and inform future research on preventing LR.

## 1 Introduction

Pancreatic cancer often presents with perivascular infiltration and invasion of the mesenteric root, involving the portal‐superior mesenteric vein (PV‐SMV) and superior mesenteric artery (SMA). This is particularly encountered in the case of pancreatic head, uncinate process, and neck cancer [1, 2]. Despite being excluded from the consideration of T stage in AJCC guideline [3], PV‐SMV invasion remains crucial in determining surgical approaches, reconstruction methods, and oncological outcome [4]. PV‐SMV involvement, along with major arteries like SMA and celiac trunk (CA), impacts resectability [5]. However, discussion on the long‐term impact of vascular structures on oncological outcomes, particularly local recurrence (LR) post curative surgery, is limited. Existing studies on recurrent patterns based on radiological features are constrained by small sample sizes [6, 7, 8], reflecting the ongoing challenge of achieving long‐term survival in advanced pancreatic cancer [9].

The primary challenge impacting the oncological outcome is the high recurrence rates following curative resection [10, 11, 12]. Unlike distant metastasis, such as liver and lung metastasis, the early detection of LR in the postoperative period poses significant difficulty using imaging modalities like Computed Tomography or Magnetic Resonance Imaging [13]. This distinction underscores the critical need for improved diagnostic strategies, such as specialized radiologists [14], to detect LR early and differentiate it from surgical complications or inflammation. Furthermore, while other image modality such as FDG PET‐CT [15] have been discussed in several studies, their clinical application and availability remain limited.

A comprehensive analysis of both clinical and radiological factors, including a detailed examination of vascular features in a larger cohort of patients undergoing pancreatectomy with or without vascular resection, is needed. Existing literature has predominantly focused on surgical techniques and outcomes in relation to postoperative vascular features. This study aims to delineate potential risk factors for LR and identify relevant vascular features in radiological images of pancreatic cancer patients undergoing pancreaticoduodenectomy (PD).

## 2 Materials and Methods

### 2.1 Patients

The study was conducted at National Cheng Kung University Hospital, a tertiary medical center in southern Taiwan. Retrospective data collection was performed using electronic records dating from December 2010 to March 2021. The study adheres to the principles outlined in the Declaration of Helsinki and received approval from the institutional review board (B‐ER‐112‐442). Inclusion criteria were as follows:

1. Pancreatic head or neck adenocarcinoma, excluding IPMN‐associated carcinoma and other subtypes such as acinar cell carcinoma, adenosquamous carcinoma, or other cell types.
2. Patients aged over 18 years, of both sexes.
3. Patients who underwent curative surgery, including PD (Whipple or pylorus preserving PD) and total pancreatectomy.
4. Patients who received either upfront surgery or neoadjuvant chemotherapy (NAC) approach. Preoperative concurrent chemo‐radiotherapy was rarely performed at our center and was not discussed in this study (only six cases were identified in this cohort).

To minimize bias, the following patients were excluded from the analysis

1. Patients with metastatic disease, either clinically or biopsy‐proven, who received conversion chemotherapy and surgery.
2. Patients with LAPC for whom resection of SMA, CA, or common hepatic artery (CHA) was indicated. This group of patients carried a significantly higher risk of surgical complications, which could complicate postoperative anatomical conditions and was therefore excluded from this study.
3. Patients with a follow‐up time shorter than 6 months, without measurable outcomes such as LR or vascular features.

### 2.2 Preoperative Evaluation

**FIGURE 1:**
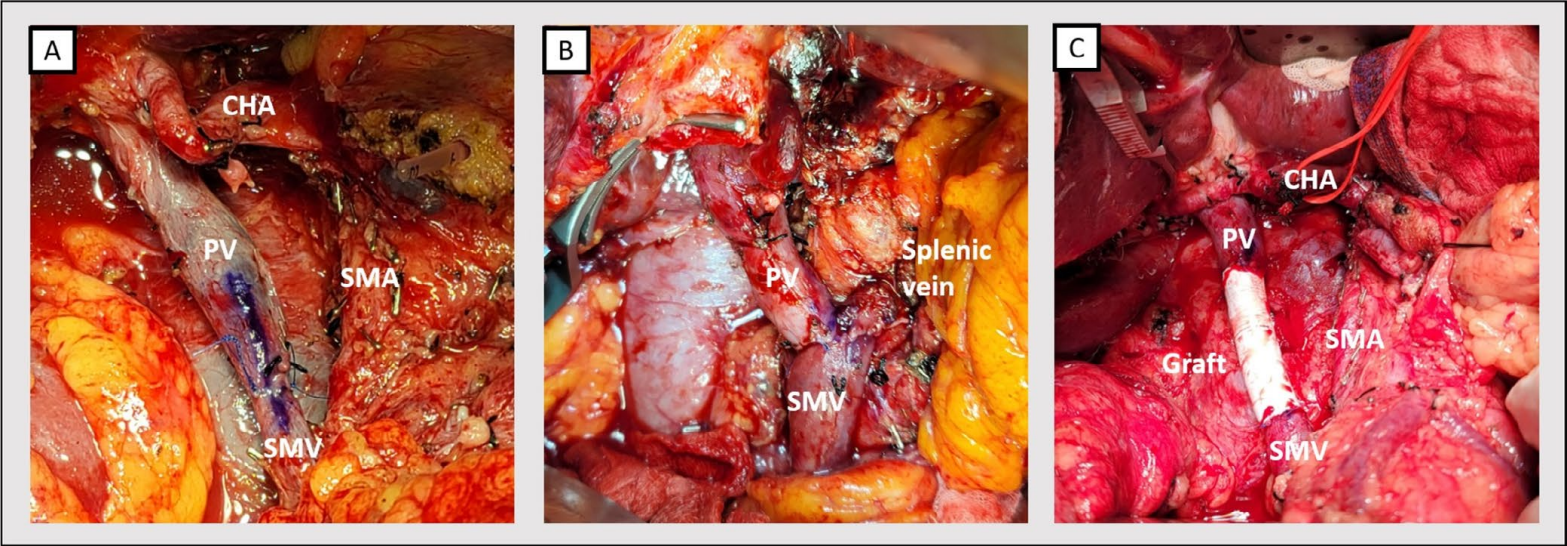
(A) Triangle surgery was performed with level II meso‐pancreas dissection. In this case the splenic vein was transected without reconstruction, and end‐to‐end anastomosis was performed for PV‐SMV reconstruction. Marking was placed at the anterior surface to prevent axis rotation, which could compromise venous flow. (B) Reconstruction of PV‐Splenic vein‐SMV was accomplished with a Y shaped anastomosis. Splenic vein reconstruction was considered only when radicality could be achieved. (C) In case with long segmental PV‐SMV resection, synthetic graft interposition was utilized for anastomosis. CHA, common hepatic artery; PV, portal vein; SMA, superior mesenteric artery; SMV, superior mesenteric vein.

Image studies were obtained primarily using abdominal computed tomography with contrast enhancement. Some patients also underwent abdominal MRI with contrast to differentiate tumor characteristics. Resectability was determined according to the eighth edition of AJCC guideline, considering invasion less than 180 or over 180° of the SMA, CA, CHA, or PV/SMV. Nakao classification [16] was utilized to simplify the magnitude of PV/SMV invasion. Level II meso‐pancreas dissection was performed for all pancreatic cancer patients. In cases of tumor invasion at the PV‐SMV junction, splenic vein preservation was not attempted (Figure 1A), and reconstruction of splenic vein was not considered for radicality. For pancreatic cancer with major artery invasion, surgery was performed only when the cancer status was well‐controlled under neoadjuvant chemotherapy and well‐discussed by multidisciplinary team.

### 2.3 PV‐SMV Reconstruction Methods

Most cases encountered segmental PV‐SMV invasion or encasement by pancreatic cancer; therefore, only two cases received wedge resection with primary repair. For segmental resection, axis marking was performed before transection to prevent rotational obstruction during vein reconstruction (Figure 1A). In a case with a resected gap below four centimeters, end‐to‐end (E‐T‐E) anastomosis (Figure 1A,B) was performed using continuous sutures with 5–0 or 6–0 Prolene in at least two threads to prevent the purse‐string effect. A biosynthetic or PTFE graft (GORE‐TEX) (Figure 1C) was considered if the gap length exceeded 4 cm or if tension was observed during reconstruction.

### 2.4 Radiological Features and Measurement

Perioperative images were independently reviewed by at least two senior doctors from the research team. The recurrence site was classified as mesenteric root, SMA, SMV, pancreas, CA/aorta, and liver hilum. The diagnosis of LR was confirmed by the presence of hypo‐vascular soft tissue tumors among the above sites, either appearing abruptly or progressively enlarging during follow‐up.

The following vascular features persisting over 3 months post‐operation were recorded for further analysis.

For PV‐SMV

1. Infiltration around the vein
2. Venous thrombosis
3. Lumen narrowing (including stenosis or total occlusion, typically involving the tumor invasion site preoperatively or at the anastomotic site in the PV‐SMV resection group)
4. Progression of angulation (the angle measured between the PV axis line and SMV axis; enlargement of the angle was defined as progression of angulation)

For SMA/CA

5 Infiltration around SMA/CA
6 Lumen narrowing (including stenosis or total occlusion).

### 2.5 Statistical Analysis

The distribution of the continuous variables was assessed by Shapiro test. Normally distributed variables were reported as mean ± standard deviation and compared using Student's *t*‐test. Non‐parametric continuous variables were expressed as median [interquartile range] and compared using the Wilcoxon rank sum test. Categorical variables were expressed as *n* (%) and compared using Pearson's Chi‐square or Fisher's exact test if *n* < 5. Statistical analysis was conducted using R software [17] version 4.2.3. Cox regression was used to analyze the risk factors for local‐recurrence free after the operation in univariable and multivariable analysis. Multicollinearity was assessed by checking Variance Inflation Factor (VIF). Survival analysis was performed by Kaplan–Meier method with packages “survival” [18] and “survminer” [19] in R. Two‐tailed *p*‐values less than 0.05, were considered statistically significant.

## 3 Results

Between December 2010 and March 2021, a total of 203 patients with pancreatic ductal adenocarcinoma were included in this study, comprising 132 cases of resectable diseases (65%), 37 BRPC (18%), and 34 LAPC (17%) at initial diagnosis. Approximately one‐third of the patients received neoadjuvant chemotherapy. Notably, although most patients who underwent concurrent PV‐SMV resection were initially classified as BRPC or LAPC (69%), 22 cases initially classified as resectable disease ultimately required more radical surgery as PV‐SMV resection.

Comparison between VR and non‐VR groups revealed that VR group was characterized by younger age (62 [53–69] vs. 65 [58–73], *p* = 0.014), a higher proportion of BRPC/LAPC (69 vs. 16%, *p* < 0.001), a greater frequency of type B–D PV‐SMV invasion (59 vs. 15%, *p* < 0.001), and a higher rate of NAC approach (54 vs. 22%, *p* < 0.001). The R0 resection rate was similar in both groups (62 vs. 66%, *p* = 0.3) but a greater number of lymph nodes were dissected in VR group (22 vs. 16, *p* = 0.009) (Table 1). The most common regimen for NAC was gemcitabine‐based chemotherapy (81%). There was no difference in the risk factors for vascular disease, including diabetes mellitus, hypertension, dyslipidemia, or the preexisting vascular disease comparing VR and non‐VR group. There were nine patients who had dyslipidemia and took statins for control. For the 19 patients with preexisting vascular diseases, 16 were coronary arterial diseases (84%).

**TABLE 1:** Clinical characteristics comparing non‐VR and VR group.

Operation method	Overall, *N* = 203^a^	Non‐VR group, *N* = 131^a^	VR group, *N* = 72^a^	*p* ^b^
Sex Female/Male	97 (48)/106 (52)	60 (46)/71 (54)	37 (51)/35 (49)	0.4
Age, years	64 [56–72]	65 [58–73]	62 [53–69]	0.014*
BMI, kg/m^2^	22.9 [21.2–25.7]	23.3 [21.2–25.8]	22.6 [21.0–25.1]	0.2
ASA III/IV	108 (53)	72 (55.3)	36 (50.4)	0.8
Diabetes mellitus	55 (27.1)	32 (24.4)	23 (31.9)	0.323
Hypertension	73 (36)	50 (38.2)	23 (31.9)	0.465
Dyslipidemia	9 (4.4)	4 (3.1)	5 (6.9)	0.351
Vascular disease	19 (9.4)	15 (11.5)	4 (5.6)	0.259
Tumor size, cm	3.0 [2.5–3.8]	3.0 [2.5–3.5]	3.3 [2.5–4.0]	0.060
Resectability when diagnosed				< 0.001*
Resectable	132 (65)	110 (84)	22 (31)	
BRPC	37 (18)	11 (8.4)	26 (36)	
LAPC	34 (17)	10 (7.6)	24 (33)	
Neoadjuvant therapy	68 (33)	29 (22)	39 (54)	< 0.001*
Type of PV/SMV stenosis*				< 0.001*
No contact	76 (37)	76 (58)	0	
Type A	65 (32)	36 (27)	29 (40)	
Type B	35 (17)	16 (12)	19 (26)	
Type C	12 (5.9)	2 (1.5)	10 (14)	
Type D	15 (7.4)	1 (0.8)	14 (19)	
Surgical margin				0.8
R0	131 (65)	86 (66)	45 (62)	
R1/R2	72 (35)	45 (34)	27 (38)	
Positive lymph node retrieved	1 [0–3]	1 [0–2]	1 [0–4]	0.12
Total lymph node retrieved	17 [11–26]	16 [10–24]	22 [14–29]	0.009*

*Note:* Vascular disease includes previous stroke or coronary arterial disease. Clinical stage was by TNM staging system according to AJCC eighth edition. PV/SMV stenosis was classified by Nakao classification. *denote statistical significance at the 5% levels, respectively.

Abbreviations: ASA, American Society of Anesthesiologists Physical Status classification system; BMI, body mass index.

^a^ Median [Q1–Q3] or frequency (%).

^b^ Pearson's Chi‐squared test; Wilcoxon rank sum test; Fisher's exact test.

Regarding adjuvant therapy, 163 patients (80%) received adjuvant chemotherapy following surgery. The proportion of patients receiving adjuvant therapy was higher in VR groups and non‐VR groups (89% vs. 76%, *p* = 0.047). The most common regimen was gemcitabine‐based chemotherapy (64.6%), followed by TS1 monotherapy (25%) and 5‐FU‐based regimen (11.6%).

### 3.1 Survival Outcome

**FIGURE 2:**
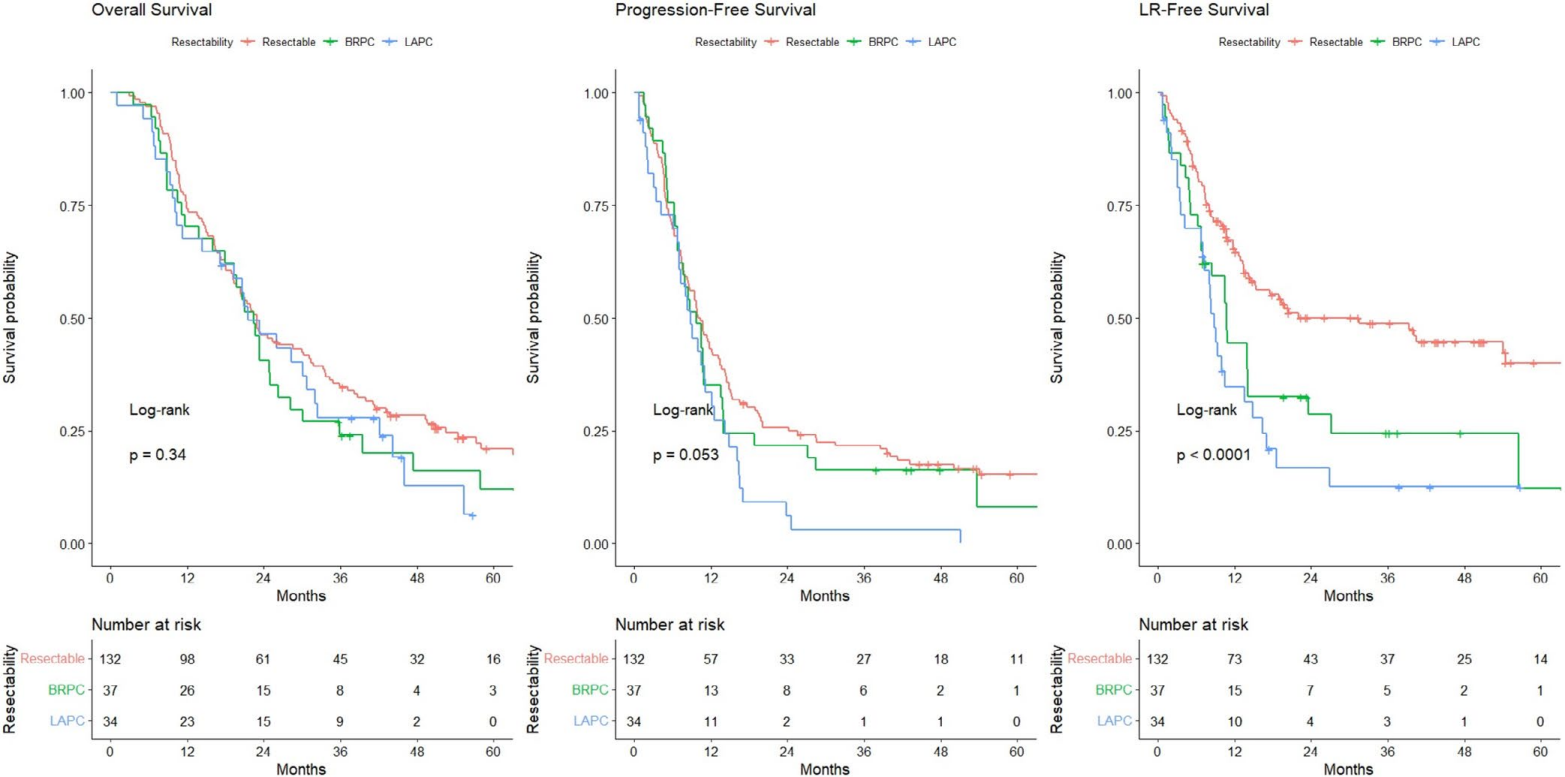
Survival analysis was conducted using the Kaplan–Meier method for overall survival, progression‐free survival, and local‐recurrence free survival, stratified by resectability: Resectable disease (red), borderline resectable pancreatic cancer (green), and locally advanced pancreatic cancer (blue). The log‐rank test was performed for comparison.

Comparison among resectable disease, BRPC, and LAPC revealed no significant difference in median overall survival (OS) by Kaplan–Meier analysis, with values of 22.9 [19.4–31.4], 22.4 [18–28.2], and 21.5 [17.2–32.5] months, respectively (*p* = 0.34). Progression‐free survival (PFS) showed slight differences in median time with borderline significance, at 10.2 [8.4–13.5], 9.7 [7.1–13.8], and 8.9 [7.0–12.6] months, respectively (*p* = 0.053). However, the LR free survival (LRFS) significantly differed among these three groups, with values of 31.5 [14.5‐NR], 10.8 [7.1–23.6], and 8.9 [7–14.9] months, respectively (Figure 2).

### 3.2 Local Recurrence Pattern

Among the 121 cases with local recurrence, 92 (76%) cases exhibited the first recurrence site as local, including five (4%) cases with simultaneous local and distant metastasis identified. The median time of LR was 8.1 months. While most cases experienced LR within 1 year after the operation, 36 (29.8%) cases experienced recurrence noticed more than 1 year later. Following the diagnosis of LR, approximately 23% of cases received salvage radiotherapy, while the remainder underwent systemic treatment as the primary rescue strategy. The site of LR occurred mostly at the mesenteric root (37%), followed by the SMA (21%) and the superior mesenteric vein (SMV) (16%) (Table 2). There was no significant difference in the recurrence pattern between the VR and non‐VR groups, except for a higher proportion receiving salvage radiotherapy (40% vs. 12%, *p* < 0.001).

**TABLE 2:** The pattern of local recurrence comparing VR and Non‐VR group.

Pattern of local recurrence	Overall, *N* = 121^a^	VR group, *N* = 47^a^	Non‐VR group, *N* = 74^a^	*p* ^b^
Local recurrence as first site, *n* (%)	92 (76)	35 (74)	57 (77)	0.7
Time to local recurrence, months	8.3 [4.5–14]	8.3 [4.3–14]	8.1 [4.1–12]	0.8
Local recurrence location				0.3
Mesenteric root, *n* (%)	45 (37)	14 (30)	31 (42)	
SMA, *n* (%)	26 (21)	12 (26)	14 (19)	
SMV, *n* (%)	19 (16)	5 (11)	14 (19)	
Pancreas, *n* (%)	12 (9.9)	6 (13)	6 (8.1)	
Celiac trunk/Aorta, *n* (%)	10 (8.3)	6 (13)	4 (5.4)	
Liver hilum, *n* (%)	9 (7.4)	4 (8.5)	5 (6.8)	
Radiotherapy for salvage	28 (23)	19 (40)	9 (12)	< 0.001*

*Note:* *denote statistical significance at the 5% levels, respectively.

^a^ Median [Q1–Q3] or Frequency (%).

^b^ Pearson's Chi‐squared test; Wilcoxon rank sum test; Fisher's exact test.

When analyzing LR patterns by gender, we found no significant differences in the overall incidence of LR between males (62%, 75/121) and females (58%, 46/82) (*p* = 0.53). However, males showed a slightly higher rate of mesenteric root recurrence (40% vs. 32%, *p* = 0.23) and venous thrombosis (11% vs. 7%, *p* = 0.36), though these differences did not reach statistical significance.

### 3.3 Risk Factors for Local Recurrence

Analysis of postoperative radiological features associated with vascular structure demonstrated a significant difference when comparing the patients with LR to those without LR (Table S1), including

1. Infiltration around PV‐SMV (61% vs. 45%, *p* = 0.024).
2. Infiltration around SMA or celiac axis (55% vs. 32%, *p* < 0.001).
3. Lumen narrowing of PV‐SMV (31% vs. 11%, *p* < 0.001).
4. Lumen narrowing of SMA or celiac axis (9.9% vs. 1.2%, *p* = 0.013).
5. Progressive angulation of PV‐SMV (19% vs. 50%, *p* < 0.001).

The thrombotic rate of PV‐SMV was higher in the patient with LR, although it did not reach statistical significance (12% vs. 6.1%, *p* = 0.14). However, given its widely reported role as a risk factor for LR, we included all six features in both univariable and multivariable analyses.

Cox regression analysis was conducted to evaluate perioperative predictors for LRFS through univariable and multivariable analysis. In univariable analysis, significant factors including resectability (BRPC, HR 1.84 [95% CI 1.18–2.88]; LAPC, HR 2.46 [95% CI 1.57–3.87]), R2 resection (HR 2.45 [95% CI 1.56–3.86]), postoperative CA19‐9 above the normal range (HR 2.44 [95% CI 1.69–3.52]), and six radiological features (*p* = < 0.001 ~ 0.003). Lymphovascular invasion (LVI) and perineural invasion (PNI) were associated with increased hazard but with only borderline significance (Table 3).

**TABLE 3:** Risk factors for local recurrence after pancreaticoduodenectomy in pancreatic cancer.

	Univariable		Multivariable	
	*p*	HR (95% CI)	*p*	HR (95% CI)
Clinical features				
Age > 65 years	0.316	0.831 (0.578–1.194)		
Male	0.655	1.085 (0.759–1.551)		
Diabetes mellitus	0.817	1.039 (0.752–1.435)		
Hypertension	0.241	0.797 (0.545–1.165)		
Dyslipidemia	0.237	0.501 (0.159–1.577)		
Vascular disease	0.710	1.120 (0.616–2.037)		
Resectability				
BRPC	0.008*	1.842 (1.176–2.884)	0.008*	2.112 (1.213–3.677)
LAPC	< 0.001*	2.462 (1.567–3.867)	< 0.001*	2.744 (1.598–4.712)
Neoadjuvant chemotherapy	0.673	1.084 (0.745–1.578)		
PV‐SMV resection	0.208	1.266 (0.877–1.827)	< 0.001*	0.405 (0.246–0.666)
Tumor size > 3 cm	0.115	1.338 (0.931–1.924)	0.952	1.013 (0.670–1.530)
Margin status				
R1 resection	0.241	1.310 (0.834–2.057)	0.867	1.041 (0.647–1.677)
R2 resection	< 0.001*	2.454 (1.559–3.863)	0.522	1.177 (0.715–1.940)
Lymphovascular invasion	0.057	1.417 (0.990–2.03)	0.555	1.132 (0.749–1.711)
Perineural invasion	0.079	1.745 (0.938–3.246)	0.142	1.688 (0.840–3.393)
Postoperative CA199 > 40 U/dL	< 0.001*	2.440 (1.694–3.515)	0.004*	1.802 (1.209–2.686)
Adjuvant chemotherapy	0.473	1.196 (0.733–1.952)		
PV‐SMV features				
Infiltration around	0.003*	1.735 (1.202–2.504)	0.325	1.248 (0.803–1.942)
Thrombosis	0.003*	2.287 (1.329–3.937)	0.029*	2.056 (1.077–3.923)
Lumen narrowing	< 0.001*	2.844 (1.92–4.214)	0.047*	1.632 (1.007–2.647)
Progression of angulation	< 0.001*	0.272 (0.170–0.433)	< 0.001*	0.314 (0.187–0.529)
SMA/CA features				
Infiltration around	< 0.001*	1.908 (1.331–2.733)	0.056	1.562 (0.989–2.467)
Lumen narrowing	< 0.001*	3.186 (1.741–5.831)	0.274	1.490 (0.729–3.045)

*Note:* Factors with *p* < 0.25 in univariable analysis were included for multivariable cox regression. Resectability: BRPC, borderline resectable pancreatic cancer; LAPC, locally advanced pancreatic cancer (Reference: Resectable disease). Tumor size: pathological tumor size. *denote statistical significance at the 0.1%, 1%, and 5% levels, respectively.

After adjusting for these variables in the multivariable analysis, R2 resection, infiltration around PV‐SMV or SMA/CA and SMA/CA lumen narrowing were no longer predictive to LR. Resectability (BRPC HR 2.11 [95% CI 1.21–3.68], LAPC HR 2.74 [95% CI 1.6–4.71]) and postoperative CA19‐9 HR 1.8 [95% CI 1.21–2.69] remained predictive, as did radiological features including venous thrombosis (HR 2.06 [95% CI 1.08–3.92]) and lumen narrowing (HR 1.63 [95% CI 1.0–2.65]). Interestingly, VR merged as a protective factor (HR 0.41 [95% CI 0.25–0.67]) against LR after adjustment. Progression of PV‐SMV angulation was also a protective factor HR 0.31 (95% CI 0.19–0.53) in both univariable or multivariable analyses.

We further examined the correlation between tumor size and vascular involvement. Among patients with tumors larger than 3 cm (*n* = 92), 47.8% (*n* = 44) required PV‐SMV resection compared to 25.2% (*n* = 28) in patients with tumors smaller than 3 cm (*n* = 111) (*p* = 0.001). This finding suggests a significant correlation between tumor size and the likelihood of vascular involvement requiring resection.

## 4 Discussion

This study presents the real‐world data regarding the LR pattern and its association with vascular features by image surveillance in a cohort of patients with right‐sided pancreatic cancer undergoing PD. Existing literature predominantly focuses on postoperative vascular features in relation to surgical techniques and outcomes, primarily addressing occlusion/stenosis [20, 21] and thrombosis [22, 23, 24, 25, 26, 27]. Some studies have combined various radiological features, but with relatively underpowered analyses [6, 8]. While previous studies have discussed these features primarily in cases involving PV‐SMV resection only, our study aimed to comprehensively analyze clinical factors alongside radiological characteristics, including infiltration of arteries and vein, stenosis, angulation, and thrombosis, in patients undergoing PD with both VR and non‐VR approaches. Despite limitations due to our single‐center retrospective design, our study sheds light on the association between perioperative vascular structural change and LR in pancreatic cancer.

Our finding revealed a LR rate of approximately 60%, aligning with previous literature reports from 40% to 84.4% [7, 13, 28, 29]. Interestingly, we observed no difference in median survival after recurrence between distant and LRs, suggesting that pancreatic cancer behaves as a systemic disease, which was also observed in the previous study [28]. This could explain the discrepancy between LRFS and PFS but not overall survival. Despite increasing utilization of neoadjuvant chemotherapy over time, we did not observe a protective effect from NAC in our study, reflecting real‐world conditions. Groot [29] reported an earlier cohort of BRPC/LAPC receiving neoadjuvant chemotherapy and showed the similar result of 80% disease recurrence. Additionally, PV‐SMV resection emerged as a protective predictor against LR in our cohort after multivariable analysis, highlighting a significant difference in baseline characteristics between VR and non‐VR groups.

The majority of LRs in our cohort occurred at the mesenteric root or SMA/SMV (totaling 74%). Kovač [7] reported the similar result as the most common recurrent site over SMA, CHA, or CA (57.4%), followed by the area near vessels (31.2%). Notably, approximately 39% in our cohort developed PV‐SMV stenosis early after the operation, often located at the anastomosis. Dynamic changes in the postoperative period, including inflammation, fluid accumulation, bowel swelling, or postoperative pancreatic fistula, may contribute to this phenomenon. The persistence of venous lumen narrowing, rather than the nearby infiltration, was predictive of LR in our data. Jaseanchiun et al. [30] reported the use of portal vein patency ratio for the patient receiving preoperative chemoradiotherapy, which also gives support to our data.

In Fujii's study [20] involving 197 patients with periampullary or pancreatic neoplasms undergoing pancreatectomy with concurrent PV/SMV resection, a follow‐up at 1 year revealed that resection of PV/SMV over 3.1 cm was associated with severe anastomotic stenosis. Contrary to this study, our series observed no association between the length of PV‐SMV resection and immediate or long‐term stenosis/occlusion, irrespective of the reconstruction method employed. Although our experience with synthetic graft reconstruction was limited, we found no increased thrombosis rate. Previous reports have documented thrombosis rates in vascular reconstruction, with Chu et al. reporting a 9.1% early thrombosis rate in PTFE graft reconstruction [22], while other studies have compared thrombosis rates between different reconstruction methods [24, 27]. A meta‐analysis [23] in 2016, incorporating 10 studies, demonstrated a superior patency rate for the primary repair group compared to synthetic grafting. With advancements in surgical techniques, there has been a shift toward utilizing autologous grafts, cadaveric allografts, and peritoneum/falciform ligament grafts, indicating the decreasing use of synthetic grafts [31]. Nevertheless, a recently updated guidelines suggested that autologous grafts, allografts, xenografts, and prosthetic grafts can all be used for vascular reconstruction (moderate weak) [32]. The surgical techniques required not only the ability to perform vascular reconstruction but also preoperative planning and intraoperative strategies to achieve a clear margin safely.

Snyder [26] reported a cohort of 120 patients undergoing PD with concurrent VR, revealing an overall thrombotic rate of 28.3%. They suggested the late thrombosis could be associated with LR. In our cohort 20 cases were observed with venous thrombus, yielding a thrombotic rate of 9.9%. Within the VR group, 10 cases developed venous thrombosis (16.1%). Despite the relatively lower overall thrombotic rate compared to Caucasian populations, we still observed the association between venous thrombosis and LR in our Asian population.

We also identified a unique radiological feature—the change in PV‐SMV angle—as a predictor for LR. In patients with liver cirrhosis, PV‐SMV angle and Splenic vein‐SMV angle were discussed in analyzing portal hypertension and development of portal vein thrombosis [33, 34]. Rutkowski et al. [35] proposed an MRI‐based modeling to study the spleno‐mesenteric confluence flow in healthy subjects and cirrhotic patients. Their finding suggested the angle between the PV‐SMV and Splenic vein‐SMV angles was highly associated with the venous flow dynamics. Despite limited evidence, this angle change may be considered one of the structural alterations following meso‐pancreas dissection and is strongly correlated with the LR. Remarkably, compared to the infiltration around major vessels, thrombosis, stenosis, and angle change emerged as the stronger predictors for LR. This discovery holds promise for enhancing the capabilities of surgical oncologists and radiologists in the early detection of LR and guiding subsequent salvage treatment strategies.

When examining the impact of gender on vascular events, our analysis revealed subtle differences that did not reach statistical significance. While males showed a slightly higher tendency toward mesenteric root recurrence (40% vs. 32%) and venous thrombosis (11% vs. 7%) compared to females, the overall LR rates were comparable (62% vs. 58%). The study by Pastrana Del Valle et al. [36] found male gender was significantly associated with increased surgical morbidities, including pneumonia, sepsis, organ/space surgical site infection; however, no vascular complication was observed in this large‐cohort analysis. The percentage of diabetes, hypertension, dyslipidemia and preexisting vascular disease were low and similar in both VR and non‐VR groups. Therefore, our data demonstrated no associations of cardiovascular risks factors to vascular complications or LR.

We also observed a significant correlation between tumor size and vascular involvement. Patients with tumors larger than 3 cm had nearly twice the likelihood of requiring PV‐SMV resection compared to those with smaller tumors (48% vs. 27%, *p* = 0.002). This correlation supports previous findings by Hackert et al. [25], who identified tumor size as an independent predictor for vascular involvement and the subsequent need for resection. However, tumor size alone was not independently associated with LR in our multivariable analysis, suggesting that vascular involvement patterns rather than tumor dimensions may be more predictive of recurrence.

Several limitations exist in our study. First, its retrospective, single‐center design may introduce selection bias and limit generalizability. Secondly, due to the limited case number, baseline characteristics matching was not performed, potentially affecting the comparability of VR and non‐VR groups. Lastly, exclusion criteria, such as cases with SMA/CA resection and those without measurable image follow‐up, may reduce the completeness of our cohort representation. Despite these limitations, our study provides a new perspective on radiological surveillance in pancreatic cancer and suggests a comprehensive approach for future clinical practices.

## 5 Conclusion

Our study provides valuable insights into the LR patterns and associated vascular features in patients undergoing PD for pancreatic cancer. Despite advancements in surgical techniques and perioperative management, LR remains a significant challenge, with mesenteric root involvement being the most common site. Our findings underscore the importance of comprehensive radiological surveillance to detect early signs of recurrence, particularly emphasizing the predictive value of unique vascular features such as PV‐SMV angle change. Furthermore, the identification of PV‐SMV resection as a protective factor against LR highlights the potential benefits of aggressive surgical strategies in selected patients. However, it is essential to acknowledge the limitations of our single‐center retrospective study and the need for further validation in larger, prospective cohorts. Ultimately, our study contributes to the growing body of evidence aimed at improving the management and outcomes of pancreatic cancer patients undergoing PD.

## Author Contributions

**Ting‐Kai Liao:** conceptualization (lead), data curation (lead), formal analysis (lead), investigation (lead), methodology (lead), software (lead), writing – original draft (lead). **Ying Jui Chao:** conceptualization (supporting), methodology (supporting), supervision (supporting), validation (equal), writing – review and editing (equal). **Wei‐Hsun Lu:** formal analysis (supporting), software (supporting), writing – review and editing (supporting). **Ping‐Jui Su:** conceptualization (supporting), data curation (supporting), validation (supporting), writing – review and editing (supporting). **Chih‐Jung Wang:** conceptualization (equal), data curation (supporting), investigation (supporting), validation (supporting), writing – review and editing (supporting). **Yan‐Shen Shan:** conceptualization (supporting), investigation (supporting), supervision (lead), validation (lead), writing – review and editing (lead).

## Disclosure

The authors have nothing to report.

## Consent

Informed consent was waived by the Institutional Review Board of National Cheng Kung University Hospital due to the retrospective nature of this study and the use of de‐identified patient data. The study was approved, IRB No. B–ER–112–442.

## Conflicts of Interest

The authors declare no conflicts of interest.

## Supporting information


**Table S1.** The radiological features of vascular structure associated with local recurrence.

## Data Availability

The data that support the findings of this study are available from the corresponding author upon reasonable request.
