# Supplementary material for: Risk Factors and Vascular Features Associated With Local Recurrence in Pancreatic Cancer Post‐Pancreaticoduodenectomy: A Retrospective Cohort Study
Source: Cancer Rep (Hoboken). 2025 Jul 1;8(7):e70267. doi: 10.1002/cnr2.70267 (PMC12210030; doi:10.1002/cnr2.70267)
Supplement: Supplementary file 1 — Table S1. The radiological features of vascular structure associated with local recurrence. [file CNR2-8-e70267-s001.docx]

Supplementary Table 1. The radiological features of vascular structure associated with local recurrence.

| **Vascular features** | Local recurrence, N=121^1^ | Without local recurrence, N=82^1^ | *p*^2^ |
| --- | --- | --- | --- |
| **Infiltration around PV-SMV** | 74 (61) | 37 (45) | 0.024 |
| **Infiltration around SMA or celiac axis** | 67 (55) | 26 (32) | <0.001 |
| **Thrombosis of PV-SMV** | 15 (12) | 5 (6.1) | 0.14 |
| **Preoperative narrowest PV-SMV diameter** | 8 [6-10] | 8.9 [5.8-10.3] | 0.3 |
| **Postoperative narrowest PV-SMV diameter** | 6 [3.9-9.0] | 7.3 [6.0-9.0] | 0.037 |
| **Lumen narrowing of PV-SMV** | 37 (31) | 9 (11) | <0.001 |
| **Lumen narrowing of SMA or celiac axis** | 12 (9.9) | 1 (1.2) | 0.013 |
| **Total occlusion of PV-SMV** | 50 (41) | 4 (4.9) | <0.001 |
| **Preoperative PV-SMV angle** | 51 [44-64] | 53 [42-64] | >0.9 |
| **Postoperative PV-SMV angle** | 52 [39-65] | 59 [49-75] | 0.007 |
| **Progression angulation of PV-SMV** | 23 (19) | 41 (50) | <0.001 |
| *^1^* Median [Q1-Q3] or Frequency (%) | | | |
| *^2^* Pearson’s Chi-squared test; Wilcoxon rank sum test; Fisher’s exact test | | | |
